# Depth-sensitive assessment of cerebral blood flow and low-frequency oscillations after traumatic brain injury in mice using time-domain diffuse correlation spectroscopy

**DOI:** 10.1117/1.NPh.13.1.015003

**Published:** 2026-01-09

**Authors:** Sahar Sabaghian, Chien-Sing Poon, Dharminder S. Langri, Timothy M. Rambo, Aaron J. Miller, Brandon Foreman, Ulas Sunar

**Affiliations:** a Stony Brook University, Department of Biomedical Engineering, Stony Brook, New York, United States; b Wright State University, Department of Biomedical Engineering, Dayton, Ohio, United States; c Quantum Opus LLC, Plymouth, Michigan, United States; d University of Cincinnati, Department of Neurology & Rehabilitation Medicine, Cincinnati, Ohio, United States; e Stony Brook University, Department of Electrical and Computer Engineering, Stony Brook, New York, United States

**Keywords:** cerebral blood flow, traumatic brain injury, time-domain diffuse correlation spectroscopy, early and late gate, optical blood flow

## Abstract

Traumatic brain injury (TBI) can lead to long-lasting impairments in cerebral perfusion, making early detection of microvascular changes critical for guiding clinical interventions. We employed time-domain diffuse correlation spectroscopy (TD-DCS) at 1064 nm to noninvasively quantify depth-resolved cerebral blood flow (CBF) and low-frequency oscillations (LFOs) in a mouse model of closed-head injury. By analyzing earlier photon arrivals (with greater superficial weighting) and later photon arrivals (with enhanced sensitivity to deeper tissue), we identified a significant drop in CBF shortly after injury, with partial recovery observed at 2 h post-trauma. Power spectral analysis of the blood flow index (BFI, a diffusion coefficient proportional to CBF) revealed significant alterations in LFO bands, particularly in slow-5 (0.01 to 0.027 Hz) and slow-3 (0.073 to 0.198 Hz) ranges. These differences, assessed using a paired Wilcoxon rank-sum test (p<0.05), were more pronounced than BFI alterations alone, indicating that LFOs may serve as sensitive biomarkers of neurovascular disruption. Our findings demonstrate the feasibility of TD-DCS for relative depth-sensitive monitoring of cerebral hemodynamics and oscillatory dynamics after TBI and highlight its potential utility in translational neurotrauma research.

## 1 Introduction

Traumatic brain injury (TBI) is a major global health concern and a leading cause of death and disability, particularly in young adults.^1^ Although primary injury occurs at the time of trauma, secondary injury processes, such as impaired autoregulation, ischemia, and neuroinflammation, can evolve over minutes to hours and significantly affect outcomes^2^ caused or exacerbated by traumatic blood flow dysregulation.^3^ Early detection of these secondary changes is essential for timely intervention and improved prognosis.^4^ Thus, continuous monitoring of cerebral blood flow (CBF) is an important measure that can be used to detect and prevent these secondary injuries.

Traditional imaging techniques such as magnetic resonance imaging (MRI), computed tomography (CT), and positron emission tomography offer detailed measurements of CBF but are costly, time-consuming, and not well suited for continuous bedside monitoring. Diffuse correlation spectroscopy (DCS) offers a promising noninvasive optical approach to monitor CBF at the bedside. DCS is a recent optical technique that provides noninvasive, continuous monitoring of CBF, especially useful for high-risk populations. DCS has shown promise in distinguishing cerebral from scalp blood flow, making it a valuable TBI monitoring.^5^^–^^15^ Compared with near-infrared spectroscopy (NIRS), DCS has higher brain sensitivity.^5^^,^^6^^,^^11^^,^^16^^–^^20^ Traditional continuous wave (CW) DCS systems face limitations in measuring absolute blood flow and can be prone to contamination coming from superficial signals such as the skull and scalp.^14^^,^^19^^,^^21^^–^^26^ Recent advancements such as time-domain diffuse correlation spectroscopy (TD-DCS)^22^^,^^27^^,^^28^ address these issues by enabling deeper tissue probing and higher signal-to-noise ratios (SNRs) using longer wavelengths, such as 1064 nm, mainly due to lower tissue scattering and higher permissible laser power.^29^^–^^31^

To investigate the early changes in a depth-sensitive manner, we applied the TD-DCS in a mouse model of TBI induced by a weight-drop. This well-established model allows the study of neurophysiological deficits and cell death associated with TBI *in vivo.*^32^^,^^33^ The first objective of this research is to quantify the early alterations in blood flow using time-domain DCS noninvasively and frequently. Growing evidence suggests that LFOs in cerebral hemodynamics, typically below 0.2 Hz, are linked to neurovascular coupling and cerebral autoregulation (CA). Previous studies have demonstrated their association with endothelial and neurogenic activity,^34^^–^^36^ whereas others highlight their sensitivity to changes in cerebral perfusion and vascular reactivity.^37^^–^^39^ More recent work suggests that alterations in specific LFO bands may serve as biomarkers of impaired neurovascular function after brain injury.^40^^–^^42^ Alterations in LFO power spectra have been linked to TBI, stroke, and other neurovascular pathologies, yet their depth-specific features remain underexplored. Thus, as our second objective, we analyzed the power spectrum density of LFOs that occur within the blood flow signal and have been linked to human and animal vascular health.^43^^–^^45^ TBI disrupts CA, damages neurovascular structures, and induces changes in functional connectivity networks that are linked by these LFOs.^46^^–^^48^ We investigated the capability of TD-DCS for detecting depth-sensitive changes in these spontaneous LFOs arising from TBI as potential biomarkers of traumatic neurovascular injury.

## 2 Results

### 2.1 Time-Domain Characteristics

**Fig. 1:**
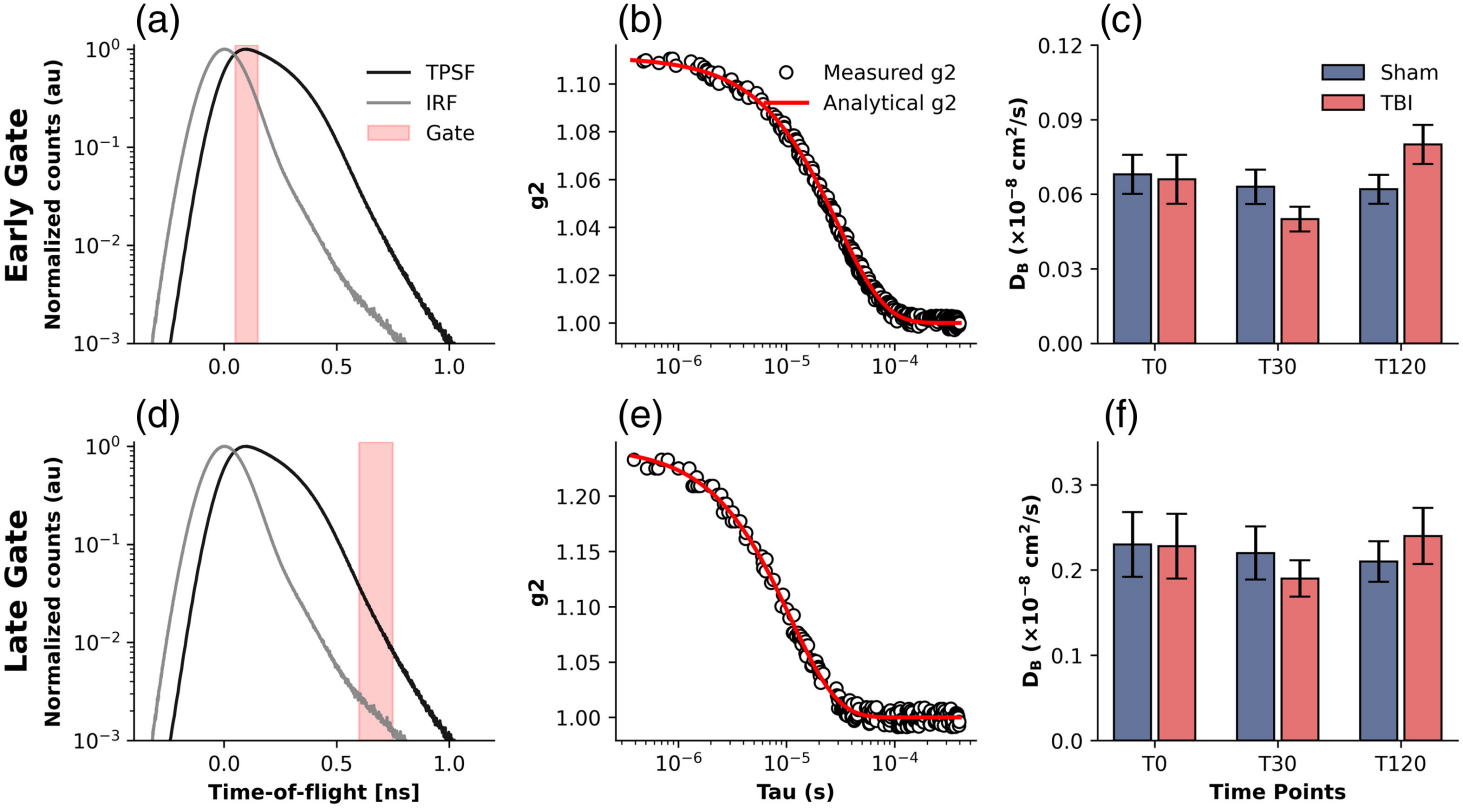
Representative TD-DCS results in early and late gates. (a), (d) TPSF overlaid with the IRF. Time zero is aligned at the IRF peak, and the shaded regions highlight the selected early (a) and late (d) gating windows. (b), (e) Representative autocorrelation curves ($g_{2}$) from the subject, shown with measured $g_{2}$ (circles) and fits using the analytical TD-DCS model (red lines) for early (b) and late (e) gates. (c), (f) Group-averaged BFI values across all mice in sham (blue) and TBI (red) groups, measured before injury (T0), 30 min after injury (T30), and 120 min after injury (T120). BFI decreased within minutes of TBI, remained suppressed for 2 h, and began to recover thereafter.

The first objective of this study was to assess the early changes in BFI obtained from three time points (i.e., T0, T30, and T120). We first examined the time-series data of BFI across all TBI mice in the early and late gates. Figure 1 summarizes representative TD-DCS results. BFI changes were tracked across three time-points (i.e., T0, T30, and T120) in both early and late gates. Overall, TBI mice exhibited a trend toward reduced BFI compared with sham animals, though these differences did not reach statistical significance (see Tables 1 and 2). As shown in the bar plots and summarized in Table 1, BFI at the late gate decreased in the TBI group from baseline (T0: $0.22\pm 0.03\times 10^{-8}\;{\mathrm{cm}}^{2}/s$) to T30 ($0.19\pm 0.02\times 10^{-8}\;{\mathrm{cm}}^{2}/s$) and gradually recovered toward baseline at T120 ($0.24\pm 0.03\times 10^{-8}\;{\mathrm{cm}}^{2}/s$). In the early gate, values were lower overall (T0: $0.06\pm 0.02\times 10^{-8}\;{\mathrm{cm}}^{2}/s$; T30: $0.05\pm 0.02\times 10^{-8}\;{\mathrm{cm}}^{2}/s$; T120: $0.08\pm 0.02\times 10^{-8}\;{\mathrm{cm}}^{2}/s$). Although these trends suggest early suppression of blood flow following TBI with partial recovery over 2 h, they did not reach statistical significance (paired Wilcoxon rank-sum test, see Table 2), likely due to the limited sample size.

**Table 1:** BFI values ($\times 10^{-8}\;{\mathrm{cm}}^{2}/s$) for the TBI group versus the Sham group at three time points, T0: baseline, T30: 30 min post-trauma, and T120: 120 min post-trauma.

Time point	Early gate		Late gate	
	Sham	TBI	Sham	TBI
T0	0.07 ± 0.01	0.06 ± 0.01	0.23 ± 0.04	0.22 ± 0.03
T30	0.06 ± 0.01	0.05 ± 0.01	0.22 ± 0.03	0.19 ± 0.02
T120	0.06 ± 0.01	0.08 ± 0.01	0.21 ± 0.02	0.24 ± 0.03

**Table 2:** Statistical results (p-values) for the changes in BFI.

Time	Early gate	Late gate
	Sham/TBI	Sham/TBI
T0	0.28	0.4
T30	0.4	0.53
T120	0.1	0.18

### 2.2 Frequency-Domain Characteristics

**Fig. 2:**
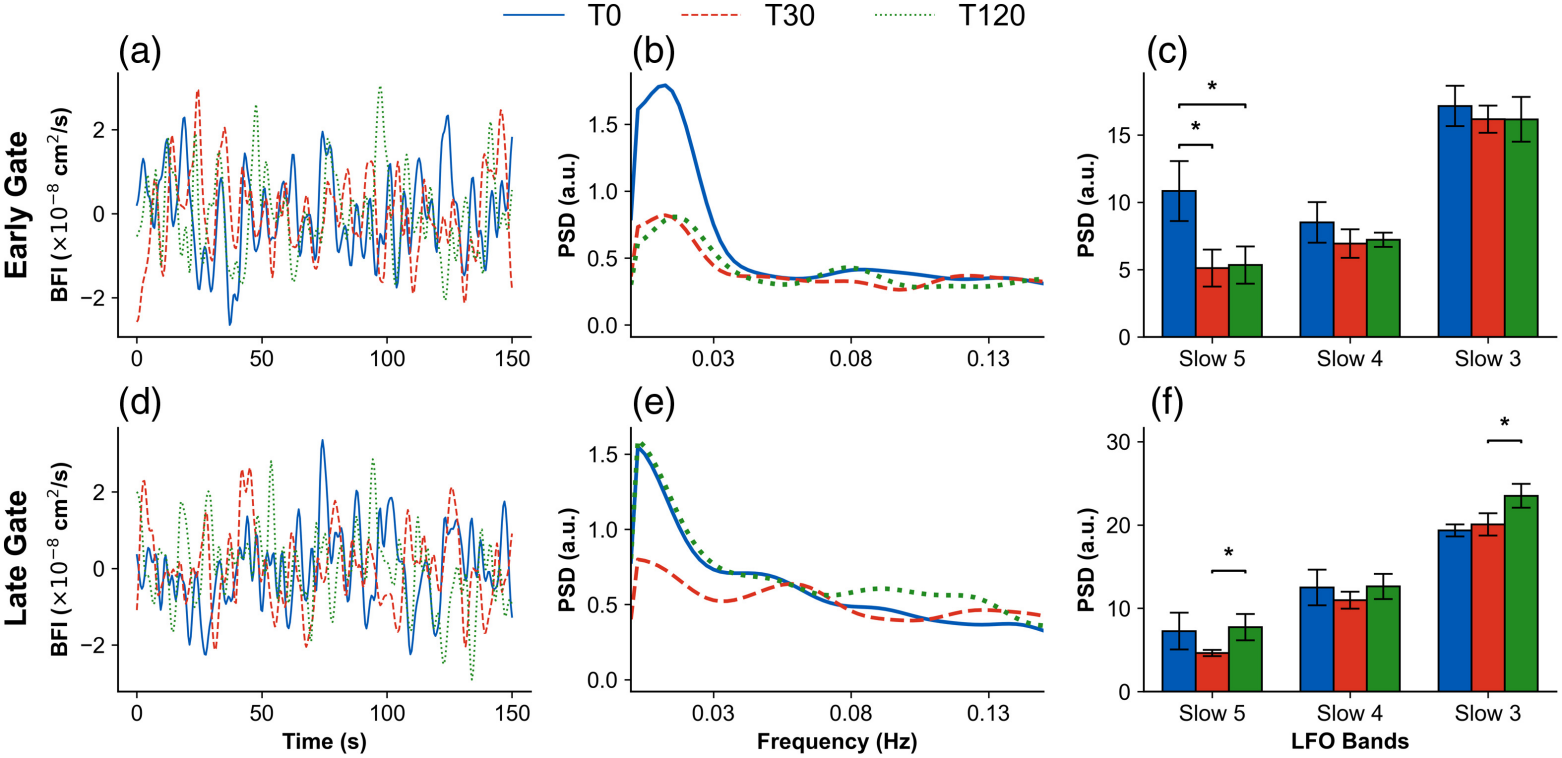
Representative BFI dynamics and PSD analysis of LFOs for the early gate (EG) and late gate (LG). Panels (a) and (d) show the group-averaged BFI time courses across all TBI subjects at baseline (T0, blue), 30 min post-injury (T30, red), and 120 min post-injury (T120, green) for EG and LG, respectively. Panels (b) and (e) illustrate the PSD spectra computed from the group-averaged BFI time courses in the low-frequency range (0.01 to 0.15 Hz), showing changes across T0, T30, and T120 for EG and LG. Panels (c) and (f) present the PSD values integrated within the three canonical LFO bands (slow-5, slow-4, slow-3) for EG and LG. Error bars represent the standard error across subjects. Statistical significance was assessed using paired Wilcoxon rank-sum tests (*p<0.05). Significant reductions in slow-5 were observed in EG between T0 versus T30 and T0 versus T120, whereas LG showed significant changes in slow-5 and slow-3 between T30 and T120.

Next, we evaluated the LFOs in tissue blood flow measured with TD-DCS for both the early gate (EG) and late gate (LG). To do this, we first calculated the group-averaged BFI time courses across all TBI data for the three measurement periods (T0, T30, and T120), after detrending and normalization, for EG [Fig. 2(a)] and LG [Fig. 2(d)]. We then quantified the oscillatory dynamics by computing the PSD from the group-averaged BFI time courses within the LFO range of 0.01 to 0.15 Hz using Eq. (7). The resulting PSD spectra for EG and LG are shown in Figs. 2(b) and 2(e), respectively. The PSD values integrated within the slow-5 (0.01 to 0.027 Hz; endothelial/autoregulation), slow-4 (0.027 to 0.073 Hz; neurogenic/brain activation), and slow-3 (0.073 to 0.198 Hz; respiratory/brain function) frequency bands are summarized for EG in Fig. 2(c) and for LG in Fig. 2(f). For the early gate [Fig. 2(c)], the baseline (T0) relative PSDs for the slow-5, slow-4, and slow-3 bands were 10.84, 8.51, and 17.16 [a.u.], respectively. At T30, these PSDs were 5.11, 6.93, and 16.18 [a.u.], whereas at T120, they were 5.34, 7.22, and 16.16 [a.u.], indicating partial recovery toward baseline. In particular, the slow-5 band showed a pronounced reduction at T30 with a modest rebound at T120, although still below baseline. For the late gate [Fig. 2(f)], the baseline (T0) PSDs for slow-5, slow-4, and slow-3 were 7.27, 12.49, and 19.34 [a.u.], respectively. At T30, these values were 4.63, 10.97, and 20.06 [a.u.], and at T120, they increased to 7.73, 12.62, and 23.50 [a.u.]. Similar to EG, the LG slow-5 band decreased at T30 and recovered at T120, whereas the LG slow-3 band exceeded baseline at 120 min. The significant p-value is indicated by * for both gates.

As Table 3 indicates, we observed that the quantifying BFI at discrete time points in both gates showed significant changes in TBI relative to sham animals. For the early-gate, the Wilcoxon rank sum test showed a significance with p-value = 0.01 for the changes between T0 and T30 and for the changes between T0 and T120 in LFO-V (band-5). For the late-gate, the rank sum test demonstrated significant changes with p-values of 0.03 and 0.04 for T30 and T120 in LFO-V and III (bands 5 and 3). Overall, as Figs 2(b) and 2(d) and Table 4 indicate, the TD-DCS system measures vasomotion tone within tissues in the VLF band to capture subtle changes in blood flow.

**Table 3:** Statistical results for LFOs. The PSD of EG shows significant changes in slow band 5 between T0/T30 and T0/T120 time-windows and for the LG through bands 5 and 3 between the T30/T120 time-windows.

Band	Early gate			Late gate		
	T0/T30	T0/T120	T30/T120	T0/T30	T0/T120	T30/T120
V	**0.01**	**0.01**	0.43	0.28	0.74	**0.03**
IV	0.25	0.25	0.19	0.52	0.67	0.22
III	0.43	0.32	0.80	0.63	0.98	**0.04**

Note: Bold values indicate statistically significant differences (*p* < 0.05).

**Table 4:** Frequency bands for low-frequency oscillations with respect to band-1 to band-5.

Band	Frequency range (Hz)	Possible mechanism
V	0.01 to 0.027	Endothelial (CA)
IV	0.027 to 0.073	Neurogenic (activation/brain function)
III	0.073 to 0.198	Respiratory/brain function (lower ends)
II	0.198 to 0.5	Mayer wavers (ABP: arterial blood pressure)/respiratory
I	0.5 to 0.75	Mayer waves (ABP)

## 3 Discussion

TD-DCS offers a powerful, noninvasive method for measuring CBF and its oscillatory features at different tissue depths. By leveraging time-of-flight (TOF) photon separation, TD-DCS enhances sensitivity to deeper cerebral hemodynamics, whereas earlier photon gates remain more strongly weighted toward superficial tissues. However, the ability of DCS is hindered by the low SNR when measuring blood flow in deeper tissue because the fraction of detected photons decreases significantly with depth.^19^^,^^22^^,^^25^^,^^49^^,^^50^ We and others have shown that SNR can be improved by performing measurements at 1064 nm using SNSPD.^30^^,^^31^^,^^51^^,^^52^ In our previous work, we demonstrated blood flow measurements of a TBI patient with 50 Hz temporal resolution in a clinical setting.^52^ In this work, we applied this approach to monitor the depth-sensitive blood flow changes in mice after injury challenge. Because of smaller skull thickness (<1  mm versus ∼10  mm in humans) and shorter source-detector separation (4 mm versus 15 mm for humans) used, the SNR was sufficient to implement narrow time-gating to provide relative separation of superficial- and deeper-weighted signals, consistent with previous work.^22^^–^^26^ Although blood flow decreased at both early and late gates, these changes did not reach statistical significance potentially due to the limited number of animals. Individual mice may also have been more strongly affected by surface impact from the weight drop, which could induce global changes. These results align with previous studies of Abookasis et al. and Witkowski et al., who also observed a similar decreasing trend =using the superficial optical speckle imaging method.^53^^,^^54^ We also reported a significant (∼15%) decrease within 30 min of continuous measurements using the speckle contrast optical spectroscopy (SCOS) method,^55^ which is originally based on the diffuse speckle correlation analysis (DSCA) method developed by the Lee group.^56^^–^^59^ Jang et al.^60^ observed an acute blood flow decrease using DCS within 30 min, whereas Buckley et al.^61^ also used DCS for monitoring repetitive concussions in mice for 24 h, monitored for several days, and observed an initial CBF decrease at 4 h.

Beyond mean blood flow, we examined LFOs in the BFI signal as these have been associated with autoregulation, vasomotion, and neurovascular coupling.^62^^–^^64^ PSD analysis revealed significant alterations in LFO bands after injury, particularly in slow-5 (0.01 to 0.027 Hz) and slow-3 (0.073 to 0.198 Hz), at both gates. These findings suggest that LFOs may be more sensitive than BFI alone in capturing the vascular consequences of TBI. Altered LFO patterns likely reflect disruptions in endothelial function, autoregulatory capacity, and vascular tone, all key components of the secondary injury cascade. Importantly, the differential LFO response in superficial versus deeper tissue underscores the value of depth-sensitive analysis.

Taken together, our results support the use of TD-DCS as a dual-domain tool. It enables both traditional blood flow quantification and frequency-domain analysis of physiological oscillations, which together may offer complementary insights into injury severity and progression. These findings align with prior reports that LFO metrics may serve as early indicators of cerebral dysfunction in TBI and other neurovascular disorders. These observations are consistent with other research indicating that TBI induces profound and sustained disruptions in blood flow, which are critical to the pathology of brain injuries. Furthermore, the differential response in blood flow and its oscillations between superficial and deeper cortical layers underscores the importance of depth-sensitive techniques in TBI research.

Despite its strengths, the current study has several limitations. The use of a mouse model, although widely used, may not fully capture the complexities and heterogeneity of TBI observed in humans. Differences in cerebral anatomy, physiology, and injury mechanisms can affect the translatability of findings. The mouse head, although a complex structure, is treated here as a homogeneous medium. The experimental results thus reflect average values of selected gates related to superficial versus deeper regions. TD-DCS has limitations in distinguishing exact contributions from different microvascular compartments within the brain compared with high-resolution imaging modalities such as microscopy and optical coherence tomography (e.g., Refs. 65–69) and mesoscopy (e.g., Refs. 70–73). Continuous monitoring could also prove beneficial for analyzing post-impact kinetics rather than three discrete time points.^54^^,^^55^^,^^60^ Furthermore, our study’s duration was confined to 120 min post-trauma to assess the early changes, whereas extending to longer periods could mimic broader clinical conditions.^61^ Our optical method can provide longitudinal monitoring over extended hours and days; thus, this aim can be pursued in future studies. Moreover, as the signal is averaged across a volume, adopting an imaging approach could enhance the assessment of specific traumatic changes at local positions, such as the impact zone versus further distance areas.^54^ Although we did not attempt to quantify optical parameters, in principle, they can be extracted using instrument response function (IRF) deconvolution methods.^74^ Future studies would benefit from integrating systemic physiological monitoring and exploring methods to enhance penetration depth and resolution to obtain a more comprehensive view of the TBI impact across the entire brain.

## 4 Conclusion

In this study, we demonstrated the utility of TD-DCS with SNSPD detection at 1064 nm for noninvasive, depth-sensitive assessment of CBF and LFOs in a mouse model of TBI. By separating superficial and deeper photon arrival times, we successfully monitored cortical hemodynamic responses to injury over time. Our findings revealed a decrease in blood flow within 30 min post-injury, followed by a partial recovery by 120 min, across both early and late gates. More notably, spectral analysis of BFI signals showed significant changes in LFO power, particularly in slow-5 and slow-3 frequency bands, indicating disruptions in vascular autoregulation and neurovascular coupling. These LFO alterations were more sensitive than BFI alone, suggesting their value as potential biomarkers for early neurovascular dysfunction after TBI. Together, this work highlights TD-DCS as a promising tool for depth-resolved, dynamic monitoring of cerebral perfusion and vascular oscillations, and lays the foundation for future preclinical and translational studies aimed at improving diagnosis and therapeutic monitoring in acute brain injury.

## 5 Materials and Methods

### 5.1 Animal Preparations and Experimental Protocols

The animal protocol was approved by the Wright State Department of Laboratory Animal Resources (LAR) and the Institutional Animal Care and Use Committee. The weight drop technique was chosen to induce TBI as it closely mimics focal head trauma, and the optical setup was used to record changes in blood flow to study the effects of TBI on CBF. The experiment involved a total of 12 female C57BL/6J mice, which were divided into two groups: four control mice and eight mice designated for the induction of TBI. Mice were anesthetized using 5% isoflurane in an anesthesia chamber for 180 s. After the initial anesthesia, mice were given 2.5% isoflurane and fitted with a nose cone to maintain sedation under anesthesia for a subsequent 180 s before starting the measurements. Baseline TD-DCS measurements were taken for the next 10 min before removing the nose cone and placing the mouse on the weight drop apparatus. In the experimental protocol, three periods were defined as baseline ($T_{-10}$: 10 min before impact), $T_{30}$ (30 min after impact), and $T_{120}$ (120 min after impact).

**Fig. 3:**
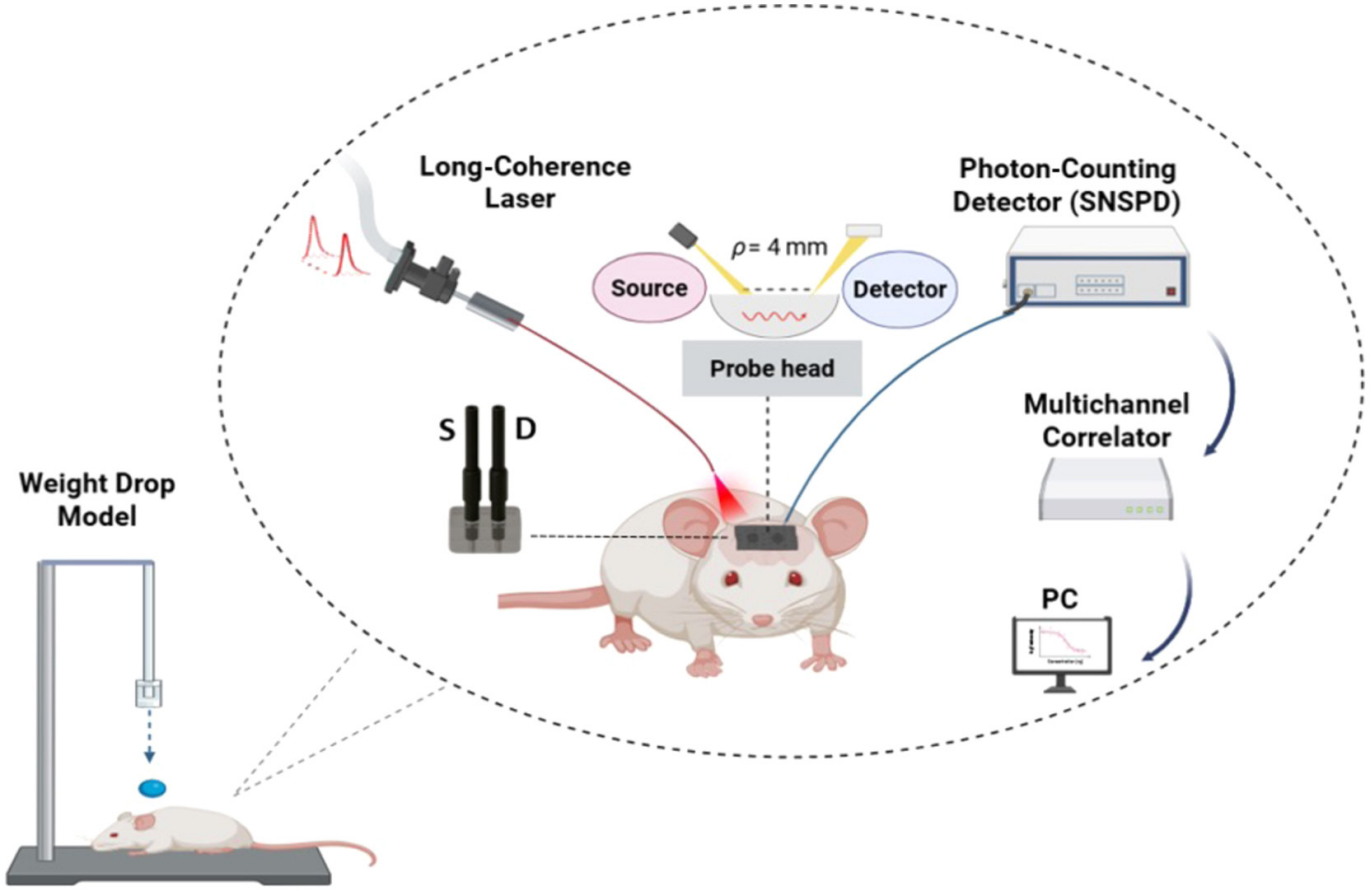
Schematic of the weight-drop TBI model and optical measurement setup. A long-coherence 1064 nm laser was coupled into a fiber-based probe positioned on the mouse scalp with a 4-mm source–detector separation. Photons were collected by a superconducting nanowire single-photon detector (SNSPD) and processed with a multichannel correlator connected to a PC to derive the blood flow index. Created with Ref. 75.

The first step of the experiment involved taking control measurements in the control group of mice. These measurements were taken for a period of 10 min to establish a baseline measurement of blood flow. The weight drop protocol^32^^,^^33^ was used to cause the TBI, which involved dropping a cylindrical metallic weight weighing 100 g from a height of 90 cm on the head of the mouse, between the anterior coronal suture and the posterior coronal suture. After inducing TBI, the time taken for the mouse to wake up was measured before transferring it back to its cage. It was then taken out of the cage and anesthetized for 5 min before the T30 timepoint. The optical measurements commenced for a duration of 10 min. The same procedure was followed at T120. The overall schematic of the weight-drop apparatus and the TD-DCS experimental setup is illustrated in Fig. 3.

### 5.2 TD-DCS System for Data Acquisition

A portable prototype of TD-DCS originally designed for clinical translation^52^ was adapted here for preclinical mouse studies. The optical probe consisted of a 600-μm-core multimode source fiber (NA = 0.39, FT600EMT, Thorlabs, Newton, New Jersey, United States) and an 8.2-μm-core detector fiber (SMF28, Thorlabs), which is few-mode at 1064 nm. The source-detector separation was ρ=4  mm. The detection fiber was coupled to a superconducting nanowire single-photon detector (SNSPD; Quantum Opus, Plymouth, Michigan, United States), providing >80% quantum efficiency at 1064 nm, <100  cps dark count rate, and ∼50  ps timing jitter. The detector output was recorded by a time-correlated single-photon counting (TCSPC) module (HydraHarp 400; PicoQuant, Berlin, Germany). The light source was a pulsed diode seed laser at 1064 nm (QLD-106P; QDLaser Inc., Kanagawa, Japan) operated at 80 MHz. Its output was amplified using a fiber-coupled optical amplifier (Mantaray-Amp-1064; Cybel LLC, Bethlehem, Pennsylvania, United States) to deliver ∼10  mW average power at the probe tip. The system IRF had a full width at half maximum (FWHM) of ∼200  ps. All measurements were performed in reflection geometry, and experiments were conducted in a dark, temperature-controlled environment (25°C) to minimize ambient noise and drift. Because the detector fiber operates in a few-mode regime at 1064 nm, the coherence factor (β≈0.2) was reduced accordingly. For each dataset, β was estimated and incorporated into the Siegert relation during curve fitting.

### 5.3 Data Processing for Blood Flow

To measure TD-DCS, we utilized two tagged time points to estimate time gate selection and to determine the intensity autocorrelation function (ACF). The first tag is the micro-time, which corresponds to the photon TOF. It is the time needed for the photon to travel from the source to the detector and is used to estimate the time-resolved photon count histogram (TPSF), also known as the distribution of time-of-flight (DTOF). The TPSF is then used to select the appropriate time gate for photon measurement. The second tag is the macro-time, which corresponds to the absolute arrival time, or the time since the measurement was started and used to determine the ACF. It is commonly defined as^22^^,^^25^^,^^49^

$$g_{2}(t_{s},\tau )={\left\langle I(t_{s},t)I(t_{s},t+\tau )\right\rangle }_{t}/{\left\langle I(t_{s},t)\right\rangle }_{t}^{2},$$ (1)

where $t_{s}$ denotes the TOF, τ is the autocorrelation delay/lag time, and $\langle \ldots \rangle_{t}$ denotes temporal averaging with respect to t distinct from $t_{s}$.

#### 5.3.1 Estimating diffusion coefficient and blood flow index

In most TD-DCS studies, the measured time-resolved intensity ACF, $g_{2}(t_{s},\tau )$ is fit to the following model (Siegert relationship):^22^^,^^25^^,^^49^

$$g_{2}^{\left(1\right)}(t_{s},\tau )=1+\beta |g_{1}^{\left(1\right)}(t_{s},\tau ){|}^{2},$$ (2)

where $g_{1}^{\left(1\right)}(t_{s},\tau )=\exp[-\xi (t_{s})\tau ]$ is the single-exponential optical field ACF of the specific path length. The symbol $\xi (t_{s})$ stands for the TOF-dependent ACF decay, and the β (0≤β≤0.5) represents the coherence factor in the Siegert relation, determined by the spatial coherence of the detected light in the experimental setup.^76^^,^^77^ Let us first assume that we know the length of the path a photon traveled, s. The optical field ACF for a single pathlength s can then be written as:^22^^,^^25^^,^^49^

$$g_{1}^{\left(1\right)}(s,\tau )=e^{-2k_{0}^{2}}D_{B}\frac{s}{l^{*}}\tau .$$ (3)

The transport mean free path $l^{*}$ is the average distance a photon travels in a scattering medium before losing memory of its initial propagation direction.^78^ It is related to the reduced scattering coefficient by $l^{*}=\frac{1}{\mu_{s}^{\prime }}$, and $k_{0}=\frac{2\pi }{\lambda }n$ is the wavenumber of light in the medium, where λ is the vacuum wavelength and n is the refractive index of tissue. $D_{B}$, the Brownian diffusion coefficient, is referred to in DCS as the BFI, a parameter proportional to CBF. The total field ACF is then obtained by integrating over all possible photon path lengths, weighted by the temporal point spread function P(s).

$$g_{1}^{\left(1\right)}(t_{s},\tau )=\int_{0}^{\infty }P(s)g_{1}^{\left(1\right)}(s,\tau )ds,$$ (4)

where P(s) is the TPSF of the tissue in a specific gate, normalized such that it is a valid probability distribution, and where the pathlength can be simply related to the TOF as $s=t_{s}*v$, where v is the speed of light in the medium. P(s) and therefore $g_{1}$ will depend on the optical properties of the medium, where the mice optical properties were estimated relative to the known phantom and used to fit the $\;g_{2}^{\left(1\right)}(t_{s},\tau )$ [Eq. (2)] to the experimentally estimated $g_{2}(t_{s},\tau )$ [Eq. (1)]. For all analyses, we assumed constant optical properties, using literature-based reference values for mouse brain tissue at 1064 nm, the absorption coefficient ($\mu_{a}=0.0122\;{\mathrm{mm}}^{-1}$) and the reduced scattering coefficient ($\mu_{s}^{\prime }=0.7\;{\mathrm{mm}}^{-1}$).^22^^,^^23^^,^^79^ Time-varying optical properties were not independently extracted from the TPSF in this study as our focus was on relative BFI changes and oscillatory features. Previous studies have shown that modest variations in optical properties do not strongly bias relative BFI trends; however, future work will incorporate TPSF-based fitting to extract dynamic $\mu_{a}$ and $\mu_{s}^{\prime }$.

In addition, we could limit our measurements to photons of known path length by time-gating to find P(s) by directly measuring the photon TOF and using the pathlength-dependent ACF $g_{1}^{\left(1\right)}(s,\tau )$ to estimate the dynamical properties, also known as the diffusion coefficient ($D_{B}$). In this study, two gates were selected: the EG (−100  ps relative to the peak of each TPSF with a width of 100 ps) and the LG (+350  ps relative to the peak of each TPSF with a width of 200 ps). The EG and LG data were autocorrelated from $5e^{-7}\;s$ to $1e^{-3}\;s$ with an integration time of 1 s.

#### 5.3.2 General expression for the ACF

The measured TOF-resolved intensity ACF $g_{2}^{\left(1\right)}(t_{s},\tau )$ [Eq. (2)] depends on the IRF and the photon TOF distribution, when experimental estimates are integrated over the TOF. Thus, under the validity of Eqs. (2) and (4), we can include those effects as follows:^20^^,^^50^

$$g_{2}^{\left(1\right)}(t_{s},\tau ,T_{\mathrm{gw}})=1+\beta |\int_{-\frac{T_{\mathrm{gw}}}{2}}^{\frac{T_{\mathrm{gw}}}{2}}P^{\prime }(t_{s},\tau )g_{1}^{\left(1\right)}(t_{s},\tau )dt_{s}{|}^{2},$$ (5)

where $T_{\mathrm{gw}}$ is the gate width, and $P^{\prime }(t_{s})$ is the normalized measured photon DTOF via a convolution (⋆) with the IRF:

$$P^{\prime }(t_{s})=\frac{1}{N_{P}}P(t_{s})⋆I_{0}(t_{s}),$$ (6)

where $N_{P}=\int_{-\infty }^{\infty }P(t_{s})⋆I_{0}(t_{s})dt_{s}$ is the normalization factor and $I_{0}(t_{s})$ denotes the IRF of the system, representing the temporal distribution of detected photons in response to an ultrashort input pulse. Therefore, to improve the SNR, the experimental estimates are integrated over the photon TOF range. Here, we only consider relatively narrow time gates, neglecting the IRF and TOF integration effects. For all reported data in this paper, $g_{2}(t_{s},\tau )$ was obtained from a rectangular time gate with 100 ps width for the early gate and 200 ps width for the late gate.

### 5.4 Power Spectral Density Analysis for LFOs

In addition to the blood flow changes, we also investigated the PSD analysis of the blood flow index to quantify the changes in LFOs. As LFOs can serve as a potentially novel metric for brain function, and optical blood flow can detect these oscillations.^35^^,^^37^^,^^55^^,^^60^ The frequency distribution of spontaneous LFOs is characterized by the following bands, labelled slow-5 (0.01 to 0.027 Hz), slow-4 (0.027–0.073 Hz), and slow-3 (0.073 to 0.198 Hz),^47^^,^^80^^–^^82^ where slow-5 can be endothelial or CA, whereas slow-4 neurogenic or brain activation, and slow-3 can be related to respiratory and brain function (lower ends like ∼0.1  Hz) mechanisms,^47^^,^^82^ as summarized in Table 4. Here, we mainly focused on the slow bands having lower frequencies (bands V, IV, and III) and presented the results related to these bands because bands I and II reflect noncortical physiology.

As more relevant to our optical approach, the PSD analysis for LFOs intensity quantification has been previously implemented in diffuse optical technologies, including NIRS^64^^,^^83^^–^^89^ and DCS.^45^^,^^90^^–^^92^ Following these approaches, LFO intensities of BFi at different time-points (i.e., baseline, 30 min after impact, and 120 min after impact) were extracted from PSDs using Welch’s method in a nonparametric approach.^93^ The PSD was calculated as

$$\mathrm{PSD}=\frac{1}{F_{s}*N_{\mathrm{seg}}}\sum |{\mathrm{FFT}}_{\mathrm{seg}}(f){|}^{2},$$ (7)

where Fs is the sampling frequency, $N_{\mathrm{seg}}$ is the number of segments, and ${\mathrm{FFT}}_{\mathrm{seg}}$ is the fast Fourier transform of the segmented signals.^93^ Briefly, the time course of the BFI data under each physiological condition (T0, T30, and T120) at the sampling rate of ∼10  Hz was first normalized and then detrended using a second-order built-in “detrend” function in MATLAB (MathWorks Inc., Natick, Massachusetts, United States) to obtain the best fit for the trend. The detrended BFI data were then transferred into a second-order zero-phase Butterworth filter with a passband at the LFO range from 0.01 to 0.15 Hz. Finally, the built-in “pwelch” function in MATLAB was used to generate the PSD over the LFO bandwidth. The PSD was calculated across all subjects (with 512 data points and 50% overlap). Then, the data were filtered into distinct frequency sub-bands to study frequency-specific CBF response.

## Data Availability

Due to privacy concerns, the supporting data cannot be made openly available. Further information and access to the data may be provided by the corresponding author upon reasonable request.
